# Formulation of a Highly Effective Inoculant for Common Bean Based on an Autochthonous Elite Strain of *Rhizobium leguminosarum* bv. *phaseoli*, and Genomic-Based Insights Into Its Agronomic Performance

**DOI:** 10.3389/fmicb.2019.02724

**Published:** 2019-12-17

**Authors:** Raquel Pastor-Bueis, Carmen Sánchez-Cañizares, Euan K. James, Fernando González-Andrés

**Affiliations:** ^1^Institute of Environment, Natural Resources and Biodiversity, Universidad de León, León, Spain; ^2^Department of Plant Sciences, University of Oxford, Oxford, United Kingdom; ^3^The James Hutton Institute, Dundee, United Kingdom

**Keywords:** common bean, Biological Nitrogen Fixation, inoculant biofertilizers, *Rhizobium leguminosarum* bv. *phaseoli*, inoculant carrier, biochar, formulation

## Abstract

Common bean is a poor symbiotic N-fixer, with a low response to inoculation owing to its promiscuous nodulation with competitive but inefficient resident rhizobia. Consequently, farmers prefer to fertilize them rather than rely on their capacity for Biological Nitrogen Fixation (BNF). However, when rhizobial inoculants are based on autochthonous strains, they often have superior BNF performance in the field due to their genetic adaptations to the local environment. Nevertheless, there is scant information at the genomic level explaining their superiority or on how their genomes may influence the inoculant performance. This information is especially important in technologically advanced agri-systems like Europe, where environmental concerns and increasingly stringent fertilizer regulations are encouraging a return to the use of rhizobial inoculants, but based upon strains that have been thoroughly characterized in terms of their symbiotic performance and their genetics. The aim of this study was to design an inoculant formulation based on a superior autochthonous strain, *Rhizobium leguminosarum* bv. *phaseoli* LCS0306, to assess its performance in the field, and to determine the genomic features contributing to the high effectiveness of its symbiosis with common bean. Plants inoculated with the autochthonous strain LCS0306 fixed significantly more nitrogen than those with the allochthonous strains *R. phaseoli* ATCC 14482^T^ and *R. etli* CFN42^T^, and had grain yield similar to the nitrogen-fertilized controls. Inoculation with LCS0306 was particularly efficacious when formulated with a carrier based upon a mixture of perlite and biochar. Whole genome comparisons revealed no differences in the classical symbiotic genes of strain LCS0306 within the symbiovar phaseoli. However, its symbiotic superior performance might be due to its genomic versatility, as it harbors a large assortment of genes contributing to fitness and competitiveness. It is concluded that inoculation with elite rhizobia formulated with perlite-biochar carriers might constitute a step-change in the sustainable cultivation of common bean in Spanish soils.

## Introduction

Common bean (*Phaseolus vulgaris* L.) is an outstanding pulse crop with more than 35 million ha cultivated per year worldwide (Mulas et al., 2011; FAOSTAT, 2019) and is a globally important source of dietary protein to millions of people (Broughton et al., 2003). Like many legume species, common bean forms root nodules in symbiosis with rhizobia belonging to different genera and species in the Alpha- and Betaproteobacteria (Peix et al., 2015). Within the Alphaproteobacteria, the species nodulating common bean mostly belong to the genus *Rhizobium* but also to other closely-related genera like *Ensifer* (*Sinorhizobium*) and *Pararhizobium* (Mousavi et al., 2015), as well as more distantly related genera like *Bradyrhizobium* (Andrews and Andrews, 2017; Mwenda et al., 2018). Dall’Agnol et al. (2013) has recently reported at least 27 species of common bean-nodulating rhizobia; these include both nitrogen-fixing and non-nitrogen-fixing strains. It has long been known that symbiotic genes, encompassing genes for plant nodulation (*nod*) and nitrogen fixation (*nif*, *fix*), are plasmid-borne in *Rhizobium* (López-Guerrero et al., 2012). Based on the phylogeny of their symbiotic genes, rhizobial strains belonging to the same species in terms of their “core” genomes are defined as symbiotic variants (symbiovars) (Rogel et al., 2014). In order to explain the multiplicity of symbiovars for a single species, it has been proposed that symbiotic genes are transferred between strains by Horizontal Gene Transfer (Andrews et al., 2018) or by mobile integrative and conjugative elements (ICEs) (Haskett et al., 2016). In the case of common bean, several nodulating symbiovars have been reported, namely phaseoli, gallicum, tropici and giardini, linked to *Rhizobium* and *Pararhizobium*, and mediterranense linked to *Ensifer* (Rouhrazi et al., 2016). Due to the numerous rhizobial partners, common bean is considered as a promiscuous legume host under field conditions (Andrews and Andrews, 2017). As a consequence of this promiscuity, common bean is often nodulated by very competitive but inefficient indigenous rhizobia (Graham, 1981; Hardarson, 1993), resulting in poor BNF, which is considered the lowest amongst the most widely grown grain legumes (Martínez-Romero, 2003).

Another consequence of the promiscuous nodulation of common bean is the inefficiency of the inoculants based on allochthonous elite strains, even when they were selected based on their reputation as good nitrogen fixers (Daza et al., 2000; Rodriguez-Navarro et al., 2000). These allochthonous strains are not successful in competition with overall inefficient native rhizobia, potentially due to their lack of adaptation to the local environment (Martínez et al., 2016). To avoid the failure of common bean inoculants, the current trend is the selection of naturally evolved locally sourced rhizobia (Díaz-Alcántara et al., 2014; Martínez et al., 2016; Koskey et al., 2017). These autochthonous symbionts show superior characteristics of competitiveness in nodule infection and occupancy due to their better adaptation to the local agro-climatic conditions (Meghvansi et al., 2010) and to their positive interaction with the resident microbial populations (Tena et al., 2016). Thus, rhizobial strains isolated under local field conditions usually result in successful inoculants, as already reported for various crops (Dall’Agnol et al., 2013), including *P. vulgaris* (Mulas et al., 2011, 2015; Yanni et al., 2016; Zhou et al., 2017).

*Rhizobium leguminosarum* bv. *phaseoli* LCS0306 (Rlp LCS0306) is indigenous to the Protected Geographic Indication (PGI) “Alubia de La Bañeza-León,” which is the region with the most ancient tradition of common bean cultivation and has the largest common bean-cropped area in Spain. Isolated from a root nodule of common bean, it was selected for its high N-fixation effectiveness under hydroponic conditions (Mulas et al., 2011). It was classified as *R. leguminosarum* on the basis of sequences of its *recA* and *atpD* genes (GenBank references JF792210 and JF792197, respectively), belongs to the symbiovar phaseoli and carries the *nodC* γ-allele present in *R. etli* Viking 1 (Mulas et al., 2011). Small-scale field trials in the PGI “Alubia de La Bañeza-León” showed that inoculation with Rlp LCS0306 produced the same grain yield as uninoculated plants given mineral Nitrogen fertilization, confirming that it was adequate for common bean inoculation (Mulas et al., 2011, 2015).

Although an adequately performing strain is an essential prerequisite in the development of successful inoculants for common bean, the non-biological components of formulations are still key bottlenecks in the commercial development of inoculants (Bashan et al., 2014). The use of pre-inoculated seeds is the most convenient delivery system, but while rhizobia survive well in inoculant formulations, many species die rapidly after seed-coating owing to desiccation (Atieno et al., 2018). Currently, the most widespread formulation consists of peat as the rhizobia carrier, plus other additives such as bacterial protectors and adhesives (Bashan et al., 2014; Atieno et al., 2018). However, the lack of natural peat deposits in several countries or their location in preserved areas, taken together with peat being a dwindling non-renewable resource, is driving the search for alternative carriers (Benrebah et al., 2007; Albareda et al., 2008). Perlite was proposed by Albareda et al. (2008) as an optimal carrier alternative to peat, as well as other mineral or organic carriers (Benrebah et al., 2007; Lugtenberg and Kamilova, 2009; Malusá et al., 2012; Bashan et al., 2014). Among them, biochar (Khavazi et al., 2007; Egamberdieva et al., 2017), and compost (Kumar and Singh, 2001; Arif et al., 2017) have been proposed as outstanding options.

Given the global importance of common bean as a crop, the improvement of its BNF capacity would be advantageous both to the environment and the economy. Currently, in the PGI “Alubia de La Bañeza-León” the BNF ability of common bean is under-used as farmers prefer to fertilize it with ammonium nitrate, which constitutes a significant financial cost (>100 Euros ha^–1^). Therefore, the general aim of this study was to design a successful inoculant for common bean based on an elite autochthonous strain with an adequate formulation which can result in grain yields that are at least equal to those obtained through current fertilization practices. The study involved first the design of the formulation based on the elite local strain *R. leguminosarum* bv. *phaseoli* LCS0306 (Rlp LCS0306) and bio-based carriers. The agronomic performance of these innovative inoculants was tested in two field trials to appraise the superiority of the inoculant containing the autochthonous Rlp LCS0306 compared to the inoculants based on the type strains of *Rhizobium etli* (Re CFN42^T^) and *Rhizobium phaseoli* (Rp ATCC 14482^T^), which are allochthonous. As the formulation based on the elite local strain Rlp LCS0306 performed better than the type strains, we then attempted to explain its superiority from a genomic perspective.

## Materials and Methods

### Common Bean Cultivar, *Rhizobium* Strains Used, and Verification of Their Nodulation Ability

Four strains were used in this study: (1) the autochthonous strain Rlp LCS0306 isolated from Sueros de Cepeda located in the PGI “Alubia de La Bañeza-León,” as described by Mulas et al. (2011), (2) *R. leguminosarum* bv. *viciae* (Rlv UPM791) (Ruiz-Argüeso et al., 1978), (3) *R. phaseoli* ATCC 14482^T^ (Rp ATCC 14482^T^) and (4) *R. etli* CFN42^T^ (Re CFN42^T^). Rlv UPM791 was included because it showed the highest similarity with Rlp LCS0306 in a genome BLAST comparison, and the other two strains were included as allochthonous controls, because they both belong to sv. phaseoli, a symbiovar that only nodulates legumes in the genus *Phaseolus* (and it is not currently possible to find strains recommended for common bean inoculation in Spain).

The common bean cultivar used was “Riñón” also known as “Riñón de León,” the most important cultivar in cropping area in the PGI “Alubia de La Bañeza – León.”

Nodulation tests were assessed in a hydroponic experiment under axenic conditions. Five plants were used per strain in independent 1 L pots, filled with sterile washed vermiculite and irrigated from a bottom reservoir with sterile N-free solution (Rigaud and Puppo, 1975). Each plant was inoculated with 1 ml of a suspension of 10^9^ cfu ml^–1^ of the corresponding strain. Five additional plants with no rhizobial suspension added were grown as uninoculated controls. The plants were grown in a growth chamber under controlled conditions (16 h light at 24°C and 8 h darkness at 18°C) for 4 weeks.

### Inoculum Production and Design of the Inoculant Formulations

The growth medium was Yeast Mannitol Agar (YMA) or broth (YMB) (Fred et al., 1928; Vincent, 1970) for the four *Rhizobium* strains. The liquid inoculum was produced in a pilot fermenter (Sartorius BIOSTAT Bplus-MO; 5 l) at 28°C and with 10% dissolved oxygen for 5 days to achieve a concentration > 1 × 10^9^ cfu ml^–1^. Following centrifugation at 8,000 *g*, the cfu ml^–1^ concentration was increased by one order of magnitude.

The individual components for the carriers were perlite, compost and biochar from pyrolysis. The compost was derived from de-alcoholized grape pomace together with vinasses of lees and lignocellulosic plant material (Supplementary Table S1). The biochar was obtained from pine bark by slow pyrolysis in a pilot plant in a semi-continuous, electrically heated reactor. The system for biochar production had an auger furnace (1,400 mm in length × 290 mm inner diameter) with three electric resistances, as described by Rosas et al. (2015). The carriers were the following: perlite (Pe) as control; compost (Co); 94% compost plus 6% biochar, denoted carbo-compost (CC); 25% perlite plus 75% biochar (PB).

To prepare the inoculum, all the carrier materials were ground, passed through an 80 μm sieve, and autoclaved in pots at 120°C for 20 min, except for the carriers with compost, which were autoclaved for 40 min. The inoculum obtained as indicated above was combined with a cell protector, consisting of 1% locust bean plus 1% trehalose (weight:volume) (unpublished data). The cellular suspension was uniformly and aseptically mixed with the carrier, according to the moisture retention characteristic curves of each carrier (data not shown). The final moisture was selected to allow a maximum volume of bacterial culture in the inoculant but providing an adequate consistency in the final mix as follows: 50% for Pe and 33% for Co, CC and PB. Therefore, the theoretical concentration of viable cells per g of inoculant after inoculation was 5 × 10^9^ cfu for Pe and 3 × 10^9^ cfu for the other formulations. After preparation, the inoculants with an available carbon source (Co and CC) were incubated for 15 days at 28°C, and then stored at 4–6°C until the sampling time, whereas those of mineral origin (Pe) or with short-time unavailable carbon sources (PB) were immediately transferred to 4–6°C.

### Determination of Bacterial Survival in the Inoculants (Shelf-Life Assessment)

The survival of the strain Rlp LCS0306 was assessed for each formulation at different time intervals (0, 60, 120, 180, 270, and 365 days after inoculum preparation). The obtained information served for a pre-selection of carriers, allowing to reject those which did not have adequate compatibility with the strain. At each sampling date, three samples for each formulation were used for the inoculum survival analysis. Viable bacteria were estimated by plating 10-fold serial dilutions on YMA plates supplemented with Congo red in duplicate for each sample. The mean values of the viable number of rhizobia per g of inoculant were then calculated for the different times and plotted on a logarithmic scale. One-way ANOVA was used to analyze the effect of the carrier in the bacterial survival at each sampling date, and Tukey test was used for *post hoc* means comparisons.

### Field Experiment

#### Experimental Design

Two field experiments within the demarcation of the PGI “Alubia de la Bañeza-León,” were conducted one in 2017 and one in 2018, in two different plots in order to preserve the principles of the existing crop rotation. The plots were more than 45 km away from the place where the strain Rlp LCS0306 was isolated. In the statistical analysis, the field experiment 2017 and 2018 respectively, were considered as the environment. The coordinates of each field, as well as the Edapho-climatic conditions and count of nodulating rhizobia based in the Most Probable Number (Beck et al., 1993), are shown in Supplementary Table S2. The sowing and harvesting dates were 16th June – 27th September, respectively, for 2017 and 12th July – 25th October 25th, respectively, for 2018, due to the abnormally high rainfall during June 2018 (Supplementary Table S2).

The experimental design followed a statistical pattern of randomized complete blocks with three replications. The experimental unit was a 49 m^2^ (7 × 7) plot, with rows 0.5 m apart and a space between plants of 0.15 m. Experimental units were spaced 2 m apart to prevent spread of rhizobia in the soil solution. The six treatments were the following six inoculants: Rlp LCS0306 formulated with the carriers Pe, P-B, Co and CC; Re CFN42^T^ formulated with Pe, and Rp ATCC14482^T^ formulated with Pe. Two uninoculated controls were also included, one fertilized with mineral nitrogen (N) and one without. Prior to sowing, seeds were dried in the shade and the appropriate quantity of seeds was then mixed with 2% by weight of the inoculant, plus 1% (weight:volume) of gum arabic solution (40% weight of gum arabic in water) as binder.

#### Agronomic Practices

Before establishing the experiment, each plot was fertilized with phosphorus (P) and potassium (K), taking into account the soil texture, the soil content of P and K, respectively, and for P, the soil pH, and in accordance with Urbano Terron (2008). The fertilizer rates were calculated for theoretical yields of 3,500 kg ha^–1^. Hence the P rates, expressed as kg P ha^–1^ were 21 kg ha^–1^ for 2017 and 24 kg ha^–1^ for 2018. All applications were as triple superphosphate (46% P_2_O_5_, that is 20% P). With regard to K, the plots received 126 kg ha^–1^ in 2017 and 131 kg ha^–1^ in 2018 as KCl (60% K_2_O, that is 50% K). The N-fertilized control plot received 170 kg N ha^–1^ which corresponds to the expected total N extraction (Urbano Terron, 2008). Nitrogen-fertilizer was applied as ammonium nitrate (27% N). Half of this amount was applied 5 days before sowing and the other half at the beginning of flowering. The fields were irrigated when necessary, according to the soil moisture content at the time using drip irrigation. The soil was kept free from weeds by mechanical systems. In 2018 the plot received lamda cyalothrin 2.5% WG (20 Days After Sowing, DAS) to control an infection of *Helicoverpa armigera*.

#### Sampling to Assess Nodulation and Recovery Rate of the Inoculated Strain From the Nodules

At the phenological stage of early pod set, R3 (one pod at maximum length), five central plants from the third row of each treatment and replication were randomly collected for nodulation assessment to appraise the number of nodules per plant and the dry nodule biomass per plant (g). In order to check the presence of the inoculated strain in each of the five plants, one random nodule was surface-sterilized, crushed in sterile distilled water, plated onto YMA and incubated at 28°C for 72 h. Following this, five isolated colonies with the morphology of strain Rlp LCS0306 were selected for DNA extraction and RAPD profiling with the M13 primer (Rivas et al., 2006), which is strain-dependent. The RAPD profile of each colony was compared with the RAPD profile of the pure strain Rlp LCS0306 (Araujo et al., unpublished).

### Sampling to Assess Nitrogen Fixation

At the phenological stage of physiological maturity, R7, eight central plants from the fifth row of each replicate plot were randomly collected and oven-dried at 70°C for 48 h. The aerial dry biomass of the common bean plants was expressed as kg ha^–1^. A sub-sample consisting on a proportional basis of the above ground biomass components was ground at 0.85 mm for ^15^N isotopic analysis. Non-legume weed species (*Sinapis arvensis* L., *Chenopodirum album* L. and *Oxalis corniculata* L.) from within the plots were collected, processed in the same way, and used as reference plants as a proxy for the ^15^N natural abundance of plant-available soil mineral N. Isotopic analysis was performed at SIDI (Universidad Autónoma de Madrid). The isotopic composition of plant samples was expressed as δ^15^N_AIR_ (‰). Raw data from δ^15^N_AIR_ (‰) are in Supplementary Table S3. The percent N derived from the fixation of atmospheric N_2_ (% Ndfa) by the common bean plants was calculated from the ^15^N abundance of the legume species and that of the non-fixing reference plant as indicated by Shearer and Kohl (1986) and Unkovich and Baldock (2008). The *B* value was determined as proposed by Pacheco et al. (2017); in the case of cv. Riñón at R7 stage this was found to be −1.97‰.

The N content in the common bean aerial biomass was calculated as: Aerial biomass N (kg N ha^–1^) = aerial dry biomass (kg ha^–1^) × N content in the aerial biomass (%); the last was determined using the Kjeldahl method. The amount of N-fixed was calculated as: N-fixed (kg N ha^–1^) = %Ndfa × Aerial biomass N (kg N ha^–1^) (Maskey et al., 2001). The soil uptake (kg N ha^–1^) was calculated as the difference between aerial biomass N (kg N ha^–1^) and N-fixed.

#### Sampling to Assess Yield and Yield Components

Sampling at harvesting was carried out in the rows that remained complete after the intermediate samplings described above, leaving at least one untouched row at each edge as a border. The six central meters of the 7th to 13th rows were hand-harvested at the harvest maturity stage. The yield was recorded as weight of air-dried beans which corresponds to the commercial grain, and then corrected to absolute dry weight after drying the seeds at 80°C to a constant weight. The dry matter of air-dried beans was 88.43%. The yield was calculated from each corresponding 21 m^2^ plot, and finally expressed as kg ha^–1^. The following yield components were also recorded for each plant: (i) number of pods per plant; (ii) number of seeds per pod; and (iii) 100-seeds dry weight in g. Finally, the harvest index (HI) was calculated on the basis of dry matter.

#### Data Analysis

##### Analysis of the inoculation treatment

The treatments considered for this analysis were the inoculation with the strains Rlp LCS0306, Re CFN42^T^ and Rp ATCC 14482^T^, formulated with perlite, plus the two uninoculated controls. The year of the experiment was considered the environment. The dependent variables were the parameters about nodulation and N fixation (Table 1 and Figure 2), yield and yield components (Table 2 and Figure 3).

**TABLE 1:** Nodulation and nitrogen symbiotic fixation indicators for the combined analysis of 2017 and 2018 and inoculant treatments in field trial.

**Inoculation treatment**	**Number of nodules per plant**	**Nodule biomass (dry) (g per plant)**	**Aerial biomass (dry) (kg ha^–1^)**	**Aerial biomass N (%)**	**Ndfa (%)**
Negative control	36.8 a	0.647 a	3428 a	2.45 a	41.3 ab
Re CFN42^T^ (perlite)	37.7 a	1.002 b	5131 bc	2.50 a	43.3 ab
Rp ATCC 14482^T^ (perlite)	29.0 a	1.154 bc	4911 b	2.53 a	46.6 bc
N fertilized non-inoculated control	34.0 a	0.512 a	5328 bc	2.35 a	39.2 a
Rlp LCS0306 (perlite)	38.3 a	1.230 c	5592 c	2.58 a	50.0 c

*The table contains the mean values for the following treatments: inoculation with the autochthonous strain *Rhizobium leguminosarum* bv. phaseoli LCS0306, the type strains of *Rhizobium etli* (Re CFN42^*T*^) and *Rhizobium phaseoli* (Rp ATCC 14482^*T*^), and the two non-inoculated controls (non-fertilized and N-fertilized). Data followed by the same letter did not significantly differ at *p* < 0.05 in the LSD test.*

**TABLE 2:** Yield components and HI for the combined analysis of 2017 and 2018 and inoculant treatments in field trial.

**Inoculation treatment**	**Pods per plant**	**Seeds per pod**	**100-seeds weight (dry) (g)**	**HI (dry basis)**
Negative control	9.31a	3.90 a	37.6 a	52.2bc
Re CFN42^T^ (perlite)	11.32b	4.30 b	37.6 a	47.6a
Rp ATCC 14482^T^ (perlite)	12.54c	4.27 b	37.8 a	50.7ab
N fertilized non-inoculated control	13.20c	4.43 b	38.9 a	54.9bc
Rlp LCS0306 (perlite)	13.40c	4.51 b	40.8 a	59.7c

*The table contains the mean values of the yield obtained for the following treatments: inoculation with the autochthonous strain *Rhizobium leguminosarum* bv. phaseoli LCS0306, the type strains of *Rhizobium etli* (Re CFN42^*T*^) and *Rhizobium phaseoli* (Rp ATCC 14482^*T*^) and the two non-inoculated controls (non-fertilized and N-fertilized). Data followed by the same letter did not significantly differ at *p* < 0.05 in the LSD test.*

The treatment factor was subjected to Analysis of variance (ANOVA) appropriate to a randomized complete block design for all the dependent variables, considering the year of the experiment as the environment. For those parameters in which the ANOVA detected significant differences, the mean values were compared using the LSD test. A pair-wise correlation analysis between the dependent variables was carried out using the Pearson coefficient.

##### Analysis of the inoculant formulation

The treatment considered for this analysis was the formulation of the strain LCS0306, with four levels, corresponding to the four different carriers, Pe, Co, CC, and PB. The data were subjected to ANOVA and the means comparison was performed with the Dunnet test, using the formulation Pe as reference for comparison. All the statistical analyses were carried out with IBM-SPSS v.24.

### Rlp LCS0306 Genome Sequencing, Annotation and Comparative Genomics

Genome sequencing was performed by MicrobesNG (Birmingham) by Illumina NGS with a coverage of 30%. The reads were trimmed using Trimmomatic (Bolger et al., 2014) and *de novo* assembly was performed using SPAdes 3.7 (Bankevich et al., 2012). Annotation was undertaken using the NCBI Prokaryotic Genome Annotation Pipeline (PGAP) v4.10 (Tatusova et al., 2016).

Average Nucleotide Identity (ANI) using MUMmer (Delcher et al., 2003) as the alignment algorithm (ANIm) or BLAST (ANIb) was calculated using the JSpeciesWS package (Richter and Rosselló-Móra, 2009). Pairwise comparisons were made between the genome sequences of the strains Re CFN42^T^ (accessions CP000133.1–CP000138.1, U80928.5), Rp ATCC 14482^T^ (accessions RJJV01000001–RJJV01000081) and Rlv UPM791 (accessions CP025505.1–CP025510.1) using a custom BLAST database on Geneious 10.0.9 (Biomatters). Clusters of orthologous groups (COGs) of proteins were predicted using the WebMGA server (Wu et al., 2011) and KAAS (KEGG Automatic Annotation Server) for the functional annotation of genes (Moriya et al., 2007). BLAST Ring Image Generator (BRIG) software was used to display circular genome comparisons (Alikhan et al., 2011).

The draft of this whole-genome shotgun project has been deposited in GenBank under the provisional accession no WNKD00000000.

## Results

### Pre-selection of the Carrier by Compatibility With the Strain Rlp LCS0306 Based on the Bacterial Survival in the Inoculant (Shelf-Life Assessment)

**FIGURE 1:**
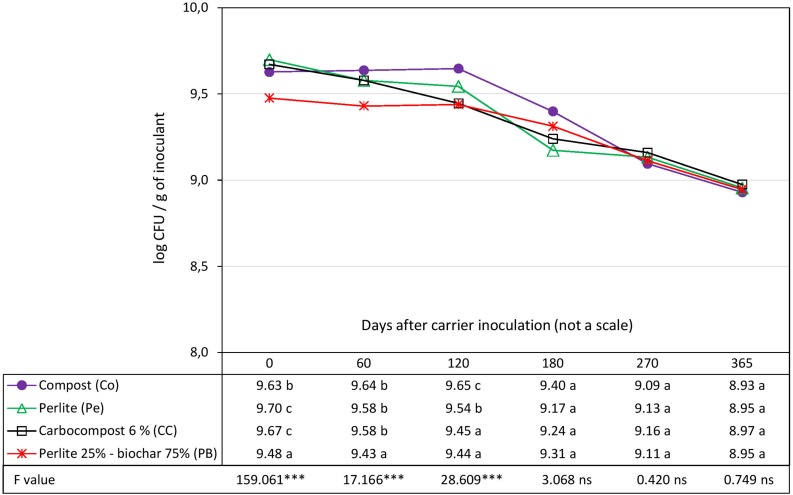
Evolution in time of the survival of Rlp LCS0306 in different carrier materials at 4–6°C. Each point represents decimal logarithmic of viable cells g inoculant^–1^ and it is the mean value of three replicas (with two independents counts per replicate). One-way ANOVA has been performed within each sampling date, thus within each column in de data table, and the *F* and significance values (^∗∗∗^*p* ≤ 0.001, ns not significant) are provided; the values followed by the same letter, within each column, are not significantly different at *p* < 0.05 in the Tukey test.

The survival of strain Rlp LCS0306 was evaluated in four different carriers: Pe as control, Co, CC and PB. The initial load of cfu g^–1^ of the inoculant was slightly but significantly higher in Pe and CC than in Co. PB showed the significantly lowest load compared to the rest of the carriers, with 0.22 logarithmic units less than Pe which had the highest value, due to the preparation process (see Inoculum Production and Design of the Inoculant Formulations) (Figure 1). All the carriers showed very similar capacities to maintain adequate survival of Rlp LCS0306, with a total loss of viability of 0.75 logarithmic units in the control (Pe), 0.70 in Co and CC, and 0.53 in PB during the whole 365 days period. At 60 days after inoculation, PB showed significantly lower load than the rest, and 120 days after inoculation, three groups were observed: The lowest load was for CC and PB, the intermediate for the control Pe, and the highest for Co. However, from 180 days onward, there were no statistically significant differences in survival in the four carriers. PB formulation showed the most stable values throughout the whole period analyzed.

### Effect of the Inoculation With Rlp LCS0306, *R. phaseoli* ATCC 14482^T^ and *R. etli* CFN42^T^ on Nodulation, Nitrogen Fixation, Yield and Biomass Production in the Field

#### Nodulation and Nitrogen Fixation Parameters

The initial nodulation assay under hydroponic conditions showed that the strain Re CFN42^T^ produced an average of 31.6 nodules per plant, significantly lower than the average number of nodules produced by Rlp LCS0306 (65.6) and Rp ATCC 14482^T^ (78.6). The latter two strains did not significantly differ in this respect (one-way ANOVA, LSD test *p* < 0.05). These strains were then evaluated under field conditions to compare their symbiotic performance according to various parameters.

In the field, the parameters evaluated were the average number of nodules, dry nodule biomass per plant and symbiotic nitrogen fixation, evaluated by the ^15^N natural abundance method. The combined ANOVA for all the parameters (year, replication and treatment) is shown in Supplementary Table S4. The treatment produced significant differences for all the evaluated parameters (*p* ≤ 0.01 or *p* ≤ 0.001, depending on parameter), except for the number of nodules per plant. There was no significant interaction between the treatment and the environment.

**FIGURE 2:**
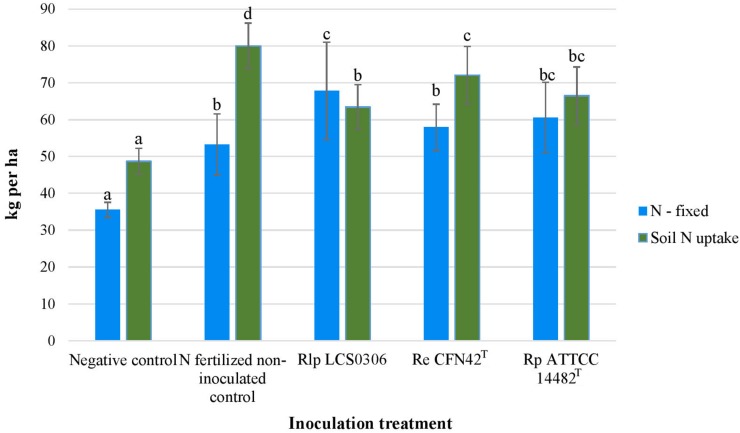
N-fixed and Soil N-uptake of the different inoculant treatments in the field trial. The figure shows the average values from the combined analysis of 2017 and 2018. Data followed by the same letter did not significantly differ at *p* < 0.05 in the LSD test.

Although unfertilized uninoculated plants were nodulated and fixed some of their N requirements, all the inoculation treatments significantly increased the amount of N fixed, particularly in those plants inoculated with strain Rlp LCS0306 which fixed nearly double the amount of N (Figure 2). The nodule biomass was significantly higher in all the inoculated treatments, although the number of nodules per plant did not show any significant difference between treatments and uninoculated controls (Table 1). The treatment inoculated with the autochthonous strain Rlp LCS0306, showed the highest values for nodule biomass (1.230 g plant^–1^), Ndfa (50%) (Table 1) and N-fixed (67.8 kg ha^–1^) (Figure 2), although these values did not statistically differ from those obtained after inoculation with Rp ATCC 14482^T^. However, Rlp LCS0306 produced significantly higher values than Re CFN42^T^ for the three parameters tested, even when Re CFN42^T^ and Rp ATCC 14482^T^ did not significantly differ between each other (Table 1 and Figure 2). As expected, the negative controls (uninoculated and non-N-fertilized) produced the significantly lowest values for the amount of N fixed (35.5 kg/ha^–1^) and the soil N uptake (48.7 kg ha^–1^), compared to the other treatments (Figure 2). The positive control (N-fertilized, uninoculated) showed the lowest Ndfa value (39.2%), differing from neither the negative control (41.1%) nor the treatment inoculated with Re CFN42^T^ (43.3%) (Table 1).

The nodule occupancy was tested in 25 independent nodules per treatment each year. Rlp LCS0306 and Rp ATCC 14482^T^ showed a high nodule occupancy both in 2017 and 2018. Rlp LCS0306 had a recovery rate from nodules of 84% in 2017 and 72% in 2018, whereas Rp ATCC 14482^T^ had a recovery of 80% in 2017 and 72% in 2018. However, for Re CFN42^T^ recovery was lower at 60% in 2017 and 44% in 2018. The bacteria isolated from the root nodules which were not the inoculated strain could be either rhizobia which actively induced nodule formation, or other endophytic bacteria which entered the nodule. In order to elucidate the identity of the other strains isolated, it would be necessary to sequence of specific genes of the unknown isolates, which is out of the scope of the present work.

#### Yield and Yield Components

**FIGURE 3:**
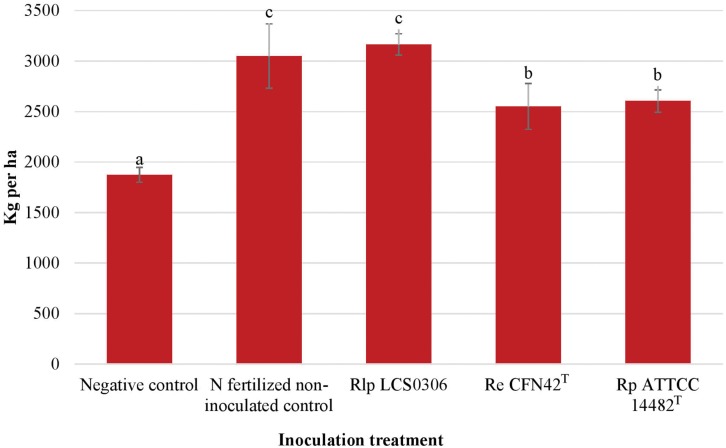
Grain yield (air dried which corresponds to 88.43% dry matter) of the different inoculant treatments in the field trial. The figure shows the average values from the combined analysis of 2017 and 2018. Data followed by the same letter did not significantly differ at *p* < 0.05 in the LSD test.

The yield and yield components were evaluated at harvest maturity (Supplementary Table S4, Table 2, and Figure 3). The combined ANOVA for all the parameters (year, replication and treatment) showed that the inoculation treatment resulted in significant increase in the grain yield, the number of pods per plant and the number of seeds per pod (*p* ≤ 0.001), but not in the 100-seeds weight. The interaction between the inoculation treatment and the year was significantly higher for the yield and all the yield components (*p* ≤ 0.001 or *p* ≤ 0.01) (Supplementary Table S4). Such interactions were due to an exceptionally good performance of Rlp LCS0306 in 2018, compared to 2017 (data not shown). Overall, inoculation with Rlp LCS0306 produced the significantly highest grain yield (3,166 kg ha^–1^), compared to the inoculation with Re CFN42^T^ (2,551 kg ha^–1^) or Rp ATCC 14482^T^ (2,604 kg ha^–1^), which did not differ from each other (Figure 3). Moreover, the grain yield value obtained for Rlp LCS0306 was similar to that from the N-fertilized positive control (3,050 kg ha^–1^) and was more than 1200 kg ha^–1^ greater than the uninoculated negative control plants (Figure 3). These significantly greater grain yields compared to uninoculated unfertilized plants were a consequence of a higher number of pods per plant and seeds per pod in all the treatments (Table 2).

#### Correlation Analysis

The parameters to estimate nodulation (i.e., nodule biomass and number of nodules) did not show any significant correlation between them (Table 3); although the number of nodules was unaffected by the inoculant treatment, the nodule biomass was. Nodule biomass was positively and significantly correlated with the %Ndfa (*R* value 0.7, *p* ≤ 0.001) and the N-fixed (*R* value 0.6, *p* ≤ 0.001), and weakly correlated with the aerial biomass (*R* value 0.53 in 2017, *p* ≤ 0.01 and 0.43 in 2018, *p* ≤ 0.05) and the grain yield (*R* value 0.50 in 2017 and 0.42 in 2018, *p* ≤ 0.05) (Table 3). The correlation coefficients (Table 3) showed that, apart from the expected positive correlation between %Ndfa and N–fixed, %Ndfa showed a weak but significant correlation with the aerial biomass (*R* value 0.47 in 2017 and 0.43 in 2018, *p* ≤ 0.05) and the grain yield, although in this case, only in 2017 (*R* value 0.51, *p* ≤ 0.05). N-fixed showed a significant correlation with the soil N uptake (*R* value 0.54, *p* ≤ 0.01 in 2017 and 0.62 in 2018, *p* ≤ 0.001), the aerial biomass (*R* value 0.88 in 2017 and 0.80 in 2018, *p* ≤ 0.001) and the grain yield (*R* value 0.83 in 2017 and 0.70 in 2018, *p* ≤ 0.001).

**TABLE 3:** Correlation (R) among: Nodule biomass, symbiotic performance, aerial plant biomass and grain yield.

**Parameters**		***R* value and significance level**	
		**Year 2017**	**Year 2018**
Dry nodule biomass (mg/plant)	Number of nodules per plant	0.342ns	0.222ns
	Ndfa (%)	0.753^∗∗∗^	0.645^∗∗∗^
	N – fixed (kg/ha)	0.619^∗∗∗^	0.627^∗∗∗^
	Soil N uptake (kg/ha)	−0.024ns	0.190ns
	Dry aerial biomass (kg/ha)	0.534^∗∗^	0.433^∗^
	Grain yield (air-dried)^1^ (kg/ha)	0.499^∗^	0.420^∗^
Ndfa (%)	N – fixed (kg/ha)	0.730^∗∗∗^	0.809^∗∗∗^
	Soil N uptake (kg/ha)	−0.163ns	0.052ns
	Dry aerial biomass (kg/ha)	0.473^∗^	0.432^∗^
	Grain yield (air-dried)^1^ (kg/ha)	0.506^∗^	0.363ns
N – fixed (kg/ha)	Soil N uptake (kg/ha)	0.541^∗∗^	0.622^∗∗∗^
	Dry aerial biomass (kg/ha)	0.877^∗∗∗^	0.800^∗∗∗^
	Grain yield (air-dried)^1^ (kg/ha)	0.833^∗∗∗^	0.701^∗∗∗^

*Significance levels: ^∗∗∗^*p* ≤ 0.001; ^∗∗^0.001 < *p* ≤ 0.01; ^∗^0.01 < *p* ≤ 0.05; ns, not significant.*

### Analysis of the Draft Genome of Rlp LCS0306

In order to correlate the agronomic traits and superior performance of the autochthonous strain Rlp LCS0306 with its genetic background, its genome was sequenced and analyzed. The draft genome of Rlp LCS0306 comprises 135 contigs, 7,395,396 bp and 60.72% GC content (Supplementary Table S5). For genospecies classification, we compared the LCS0306 genome to the representative strains of *R. etli, R. phaseoli* and the closely related strain, Rlv UPM791. The highest ANI scores were obtained against Rlv UPM791 (Table 4, ANIm 98.19%, ANIb 97.39%). As genomes that belong to the same species show genomic ANI values above 95%, the obtained ANI values indicated that Rlp LCS0306 and Rlv UPM791 were members of the same genospecies.

**TABLE 4:** Average nucleotide identity (ANI) comparison.

	**Rlp LCS0306**	**Rlv UPM791**	**Rp ATCC 14482^T^**	**Re CFN42^T^**
**ANIm**				
Rlp LCS0306	^∗^	98.19% [87.14%]	89.06% [65.03%]	88.78% [63.86%]
Rlv UPM791	98.19% [82.87%]	^∗^	88.27% [57.89%]	87.96% [56.97%]
Rp ATCC 14482^T^	89.06% [72.60%]	88.27% [67.75%]	^∗^	90.42% [75.80%]
Re CFN42^T^	88.78% [72.97%]	87.96% [67.98%]	90.41% [77.59%]	^∗^
**ANIb**				
Rlp LCS0306	^∗^	97.62% [86.96%]	86.80% [66.73%]	86.45% [66.57%]
Rlv UPM791	97.39% [82.72%]	^∗^	85.64% [60.59%]	85.17% [60.77%]
Rp ATCC 14482^T^	87.32% [73.05%]	86.28% [69.09%]	^∗^	88.86% [76.26%]
Re CFN42^T^	87.07% [73.53%]	85.87% [70.48%]	89.01% [77.64%]	^∗^
**TETRA**				
Rlp LCS0306	^∗^	0.99956	0.99728	0.99725
Rlv UPM791	0.99956	^∗^	0.99631	0.9969
Rp ATCC 14482^T^	0.99728	0.99631	^∗^	0.99904
Re CFN42^T^	0.99725	0.9969	0.99904	^∗^

*Values represent the aligned percentage and correlation indexes of their pairwise comparisons: ANIm (ANI using MUMmer), ANIb (ANI algorithm using BLAST), and Tetra-nucleotide signatures. ANI scores obtained from JSpeciesWS with the following genomes: *R. etli* CFN42^*T*^ (Re CFN42^*T*^) (6,530,093 bp; 7 molecules); *R. phaseoli* ATCC14482^*T*^ (Rp ATCC 14482^*T*^) (6,652,103 bp; 81 contigs); *R. leguminosarum* bv. v*icieae* UPM791 (Rlv UPM791) (7,837,567 pb; 6 molecules); *R. leguminosarum* bv. *phaseoli* LCS0306 (Rlp LCS0306) (7,395,396 pb; 135 contigs).*

The Cluster of Orthologous Groups (COG) analysis reflected a large number of protein families involved in metabolism (Supplementary Table S6). The metabolic network of LCS0306 was constructed by the KEGG automatic annotation server KAAS, confirming that LCS0306 resembles Rlv UPM791, Re CFN42^T^ and Rp ATCC 14482^T^ in terms of central metabolism. All the strains harbor the genes encoding the enzymes of the tricarboxylic acid (TCA) cycle, required for aerobic respiration and energy production; the pentose phosphate pathway, required for the oxidation of glucose and the synthesis of nucleotides, and the Entner–Doudoroff pathway, for the catabolism of glucose to pyruvate. These similarities were reflected in the growth pattern with different carbon sources (Supplementary Table S7). A noticeable difference in this assay was the assimilation of a higher number of both carbon and nitrogen sources in the case of LCS0306, which combined the metabolic abilities of Re CFN42^T^, Rp ATCC 14482^T^ and of the strain of *R. leguminosarum* tested, USDA 2370^T^, thus highlighting the metabolic versatility of the autochthonous strain.

The ability to persist in the soil and outcompete local rhizobia populations is based on many different parameters. At the genomic level, different traits have been described as having a role in competitiveness, such as motility and chemotaxis, exopolysaccharide (EPS) production, ABC transporters or secretion systems among others. Some of these traits have been analyzed in Rlp LCS0306 to give an overview of its genomic potential in terms of competition (Supplementary Table S8) and symbiosis (Supplementary Table S9).

Given the proposed role of secretion systems in rhizosphere colonization ability, the secretion systems of the strains were also analyzed in order to explain their competitiveness (Supplementary Table S8). Strain Rlp LCS0306 contains a large repertoire of secretion systems that combines those of Re CFN42^T^, Rp ATCC 14482^T^ and Rlv UPM791. For instance, Rlp LCS0306 contains the T1SSd proteins orthologous to the PrsD and PrsE proteins required for biofilm formation. The Rlp LCS0306 genome also harbors a putative T4SS-pili (*virB1–virB11*) system homologous to the cluster in pRlvA, involved in colonization of surfaces in Gram-negative bacteria. Strains Rlp ATCC14482^T^ and Rlp LCS0306 harbor syntenic *imp* (*tss*) and *hcp* clusters encoding a putative T6SS. As with Re CFN42^T^, Rlp LCS0306 contains a homologous T3SS cluster that might also play a role in symbiosis, and the T4SS *traGDCAFBHMR* genes involved in conjugal transfer, followed by the *nocQMT* nopaline transporter, a signal involved in DNA transfer.

**FIGURE 4:**
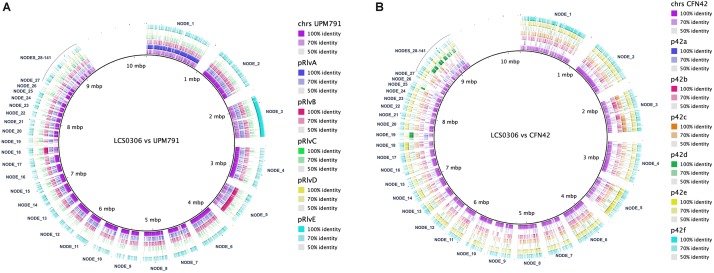
Genome comparison between Rlp LCS0306 and Rlv UPM791 and Re CFN42^T^. The figure shows the global synteny between the chromosome and plasmids from the fully closed genomes of Rlv UPM791 **(A)** and Re CFN42^T^ **(B)** against the contigs of Rlp LCS0306, labeled in the alignment.

In *Rhizobium*, the symbiotic genes, i.e., the genes involved in nodulation and nitrogen fixation are plasmid-borne. When the Rlp LCS0306 genome was aligned against the genome of Rlv UPM791 (Figure 4A), the Rlp LCS0306 contigs exhibited not only a high degree of synteny with the chromosomal sequence, but also with the chromid pRlvA and plasmids pRlvB and pRlvE, whereas pRlvD was absent and pRlvC showed a very low sequence conservation. The Rlp LCS0306 genome sequence was then compared against that of Re CFN42^T^ (Figure 4B); the Rlp LCS0306 contigs showed homology to the symbiotic plasmid of Re CFN42^T^ (p42d), indicating that Rlp LCS0306 contained a putative symbiotic plasmid belonging to the symbiovar phaseoli. As expected from its high efficiency in N-fixation, Rlp LCS0306 harbors all the nodulation and nitrogen fixation genes required to establish a successful symbiotic relationship with *Phaseolus* (Supplementary Table S9).

### Effect of the Formulation of the Inoculant Containing Strain Rlp LCS0306 on Nodulation, Nitrogen Fixation, Yield and Yield Components of Field-Grown Common Bean

In consideration of previous inoculant formulation designs and the symbiotic performance parameters of Rlp LCS0306, the formulation of the LCS0306-containing inoculant was tested under agronomically realistic conditions in the field. The combined ANOVA for all the parameters combined (year, replication and formulation treatment) is shown in Supplementary Table S4. The replication parameter did not produce any significant difference in the dependent variables, and the interaction between replication and treatment was weakly significant only for two parameters. Interestingly, the formulation produced significant differences for the amount of N-fixed, soil N uptake (*p* < 0.05 in both cases), grain yield, pods per plant and 100-seeds weight (*p* < 0.01). There was a significant interaction between the formulation and the year for 100-seeds weight (*p* < 0.01) due to the fact that better results were achieved in 2017 for CC formulation and in 2018 for PB formulation.

The formulations with biochar produced significantly higher values for the following parameters, compared to the control (Pe) (Table 5): PB formulation produced significantly higher values for soil N uptake, grain yield and pods per plant (*p* < 0.01 in the Dunnet test). Moreover, CC formulation produced significantly higher number of pods per plant and 100-seeds weight (*p* < 0.05). The best performance of the Rlp LCS0306 inoculant was obtained with the PB formulation, with a 15% higher yield compared to the perlite control.

**TABLE 5:** Field evaluation of different formulations of the autochthonous strain Rlp LCS0306 from *Rhizobium leguminosarum*.

**Formulation of the inoculant**	**Nodule number per plant**	**Nodule biomass (dry) (g per plant)**	**Aerial biomass (dry) (kg/ha)**	**Ndfa (%)**	**N fixed (kg/ha)**	**Soil N uptake (kg/ha)**	**Grain yield (air dried)^1^ (kg/ha)**	**Pods per plant**	**Seeds per pod**	**100-seeds weight (g)**	**HI (dry basis)**
Control (Pe)	38.3	1.230	5592	50.0	67.8	63.4	3165	13.40	4.51	40.8	54.6
CC	31.0ns	1.099ns	5509 ns	48.5ns	67.2ns	68.4ns	3281ns	14.22^∗^	4.40ns	41.8^∗^	57.2ns
Co	33.5ns	1.081ns	5588 ns	45.5ns	63.5ns	73.3ns	3185ns	13.76ns	4.52ns	39.9ns	55.0ns
PB	34.5ns	1.189ns	6093 ns	49.3ns	81.0ns	79.9^∗∗^	3640^∗∗^	14.48^∗∗^	4.65ns	41.5ns	57.3ns

*The table includes the mean values of the parameters related with nodulation, nitrogen fixation, yield, yield components and HI for the combination of years and treatments. ANOVA values are in Supplementary Table S4. The formulation differed in the carrier, with the following options: Control consisting on perlite (Pe); compost (Co); compost 94% in weight plus biochar 6% in weight named as carbocompost (CC); perlite 25% in weight plus biochar 75% in weight (PB). For each dependent variable, mean values were compared to the control formulation with the Dunnet test, with the following significance levels: ^∗∗^0.001 < *p* ≤ 0.01; ^∗^0.01 < *p* ≤ 0.05; ns not significant. ^1^Corresponds to the commercial beans (11.57% dry matter).*

## Discussion

Despite the advantages of BNF and other microbial processes in agriculture, the use of bacterial inoculants to provide nutrients to crops or to promote their nutrient use efficiency, tolerance to abiotic stress, or crop quality, is increasing but still not a common practice (Bhardwaj et al., 2014). Especially in developed countries, the easy availability of N-containing mineral fertilizers and the perceived erratic field performance of inoculants, have discouraged farmers from using them (Stamenković et al., 2018; Barquero et al., 2019). In the case of Europe, new rules about fertilizers have been recently approved, such as Regulation (EU) 2019/1009 which pays special attention to the regulation of the use of microbial inoculants targeted to improve crop nutrition. Thus, a surge into the market of this kind of products is to be expected. To avoid the aforementioned erratic performance of inoculants which could generate a significant failure, it is necessary to develop elite inoculants capable of satisfying the increased demand by markets in developed countries. For nitrogen-fixing rhizobia, research must focus on three main aspects: the intrinsic characteristics of the strain, the formulation, and the optimization of the production process by industrial fermentation (Herridge et al., 2008; Herrmann and Lesueur, 2013; Bashan et al., 2014; Checcucci et al., 2017). The present work has focused on the analysis of the determinant genetic factors of the strain, its symbiotic performance and the optimal inoculant formulation, with the aim of designing a successful inoculant for common bean based on autochthonous strains with an adequate formulation explained from agronomic and genomic perspectives.

Regarding the strain, one of the main challenges is the selection of superior rhizobial strains by inferring a high performance only based on their genetic features (Checcucci et al., 2017; Aguilar et al., 2018). It is necessary to separately consider colonization and nodulation abilities from symbiotic nitrogen fixation abilities (Checcucci et al., 2017), as rhizobial strains highly competitive for nodule occupancy do not necessarily fix nitrogen efficiently (Westhoek et al., 2017). Thus, elite strains must combine the two aforementioned capabilities, i.e., effectively compete with the native rhizobia for nodule occupancy and effectively provide the plant with fixed nitrogen (Checcucci et al., 2017; Onishchuk et al., 2017).

The strain Rlp LCS0306 was pre-selected among several isolates on the basis of its N-fixation effectiveness in axenic conditions (Mulas et al., 2011), which optimizes the interaction between the bacterial and plant genotypes in terms of N-fixation (Sessitsch et al., 2002). In the present study, strain Rlp LCS0306 has proven to be a superior strain in the field trial, as shown in terms of symbiotic efficiency (Ndfa, total N-fixed) and most importantly, in grain yield, which was increased by more than 1200 kg ha^–1^ above uninoculated plants. As symbiotic N-fixation abilities are uncoupled from colonization and nodulation abilities (Westhoek et al., 2017), the high performance of Rlp LCS0306 in the field must be due to the fact that, in addition to being a good N-fixer, it is also competitive. This ability has indeed been confirmed by the high recovery of the inoculant from the sampled nodules compared to the soil-borne rhizobia.

### Competing Ability of the Strains Used as Inoculant Treatments

Rhizobia are ubiquitous in the soil, their ability to form nodules in the presence of other strains determine their nodulation competitiveness (Onishchuk et al., 2017). In our field experiment, the resident rhizobia were capable of forming root nodules, which is a common situation for this promiscuous crop (Andrews and Andrews, 2017). Interestingly the number of nodules per plant was similar in the un-inoculated controls and in the inoculated treatments regardless of the strain used as inoculant treatment, which is consistent with the tight control which the plant exerts on the number of nodules (Ferguson et al., 2019). However, the nodule biomass differed between treatments and controls, being significantly higher in the inoculated treatments compared to the uninoculated controls. Moreover, the recovery of the inoculated strains reached the highest value for Rlp LCS0306 followed by Rp ATCC 14482^T^. The inoculated strain was recovered in over 75% of the nodules, whereas Re CFN42^T^ was recovered in only 52% of the nodules of the treatment inoculated with this strain. Indeed, the three strains tested overcame in competitiveness the soil resident rhizobia to varying degrees. Therefore, even if nodule characteristics such as size and biomass partitioning are strongly influenced by the common bean genotype (Rodiño et al., 2011), the rhizobial strain plays an important role in the nodule characteristics, as plants can sanction nodules that are inefficient at fixing nitrogen (Kiers et al., 2003), resulting in inefficient nodules of smaller size (Westhoek et al., 2017). In our experiment, soil-borne strains, which are less efficient than the inoculated ones, have produced smaller nodules, resulting in the observed differences in nodule biomass. Thus, in order to evaluate the nodulation success of a given strain, the number of nodules alone is not a definitive parameter and should be considered along with the nodule biomass and the retrieval of the inoculated strain in the nodules produced by the legume host.

In order to explain competitiveness from a genomic perspective, the presence of secretion systems in Plant Growth Promoting Rhizobacteria (PGPRs) and rhizobial strains has been proposed to play a role in their rhizosphere colonization ability (Gupta et al., 2014). The T3SS, T4SS, and T6SS are generally used to inject effector proteins directly into eukaryotic host cells or into other bacteria, which can mediate compatibility with the host in rhizobia (Nelson and Sadowsky, 2015). Accordingly, Rlp LCS0306 contains a large repertoire of secretion systems, which could explain its competitiveness in field conditions. Strains Rlp LCS0306, Rp ATCC 14482^T^, Rl Norway and Rlv 3841 harbor syntenic *imp (tss)* and *hcp* clusters encoding a T6SS (Liang et al., 2018; Sánchez-Cañizares et al., 2018). Impaired T6SS mutants in *R. etli* Mim1 have been shown to generate small and white nodules in *P. vulgaris*, although with similar nitrogenase activity. The authors suggested a positive role for T6SS in high competition with other soil bacteria, as it was active at high cell density and in the presence of plant exudates (Salinero-Lanzarote et al., 2019). Rlp LCS0306 also harbors the T4SS-pili present in Rlv UPM791 and a putative T3SS, absent in the reference strains Rlv UPM791 and ATCC14482^T^. Rhizobia with a functional T3SS (*R. etli* CFN42^T^, *S. fredii* HH103, *B. diazoefficiens* USDA110, or *Rhizobium* sp. NGR234) secrete nodulation outer proteins (Nops) in the presence of flavonoids, inducing the transcription of nodulation genes (Jiménez-Guerrero et al., 2017).

Competition has also been discussed from two different perspectives by Onishchuk et al. (2017): exploitative (indirect), involving more effectively utilizing a common limiting nutrient, or by interference (direct), preventing other cells from growing and surviving in the environment. Regarding exploitative competition, bacterial chemotaxis toward exuded compounds is an important trait for root colonization and plant-driven selection of microorganisms (Bais et al., 2006). In particular, the major chemotaxis gene cluster of *R. leguminosarum* bv. *viciae*, Che1, present in Rlp LCS0306, has shown to be essential for competitive nodulation (Miller et al., 2007). The diversity and variety of transport systems in rhizobia reflects the nutritional complexity of the rhizosphere environment (Prell and Poole, 2006) and are, therefore, important for growth and exploitative competition. Accordingly, the Rlp LCS0306 genome contains 183 genes involved in putative ABC transporters, such as the ABC-type broad specificity amino-acid transporter *aapJQMP*, upregulated in bacteroids of both dwarf bean (*P. vulgaris*) cv. Tendergreen (Green et al., 2019); *teuBAC1C2*, required for utilization of root exudates (Rosenblueth et al., 1998), or *nocQMT* and its regulator *nocR*, an uptake ABC transporter for nopaline, which may confer competitive ability (Oger et al., 1997).

In terms of interference competition, one of the strategies is the production of antibacterial compounds, such as bacteriocins (Onishchuk et al., 2017). Production of small bacteriocin appears to be a typical character of all fast-growing rhizobia (*R. leguminosarum*, *R. trifolii* and *R. phaseoli*) (Wijffelman et al., 1983). The quorum sensing system *cinRIS*, responsible for its production (Schripsema et al., 1996) and present in *R. leguminosarum* strains (e.g., 3841, UPM791) and Rp CFN42^T^ (Wisniewski-Dyé and Downie, 2002) is also conserved in Rlp LCS0306.

### Genomic Features Related to the Superior Field Performance of Rlp LCS0306

Although all of the inoculated strains produced higher yields than the native ones, the particularly high N-fixing ability of Rlp LCS0306 in the field has been demonstrated, i.e., Ndfa of 50%, compared to 40% for native rhizobia, and an almost doubling of total N-fixed. Moreover, the inoculation with the strain Rlp LCS0306 produced the same aerial biomass and grain yield as the N-fertilized control. Thus, this autochthonous strain produced considerably higher aerial biomass and yield than the resident strains, and the yield was even higher than in the treatments inoculated with the other strains, hence confirming the agronomic potential of Rlp LCS0306. According to the Observatory of prices of agriculture and livestock products of Castille and León (Spain), the medium sale price of dry beans in the PGI “Alubia de La Bañeza-León” was 100.54 eur 100 kg^–1^ for 2017 and 2018. Thus, in our field trials the increase in the gross income due to the inoculation would have been 1,245 eur in 2017 and 1,352 eur in 2018. Therefore, the present study has indicated that rhizobial inoculation with elite strains like Rlp LCS0306, if applied, could constitute a step-change in the sustainable cultivation of common bean in Spanish soils.

However, the improvement of the grain yield or the aerial biomass produced by the crop as a consequence of inoculation, was only partially explained on the basis of the Ndfa (%) or the Nodule biomass, i.e., the correlation between aerial biomass or the grain yield with the Ndfa or the Nodule biomass was, at the most, weakly significant and not in all the cases there was statistical significance. The aforementioned results indicate that even if the functional link between plant growth and symbiotic functioning proposed by other authors (Belane and Dakora, 2010; Qureshi et al., 2013; Mohale et al., 2014) has been reflected in our experiment, it is not enough to fully explain the positive effect of Rlp LCS0306 inoculation on the crop yield. Indeed, the Ndfa value of 50% indicates that even with a superior strain like Rlp LCS0306 the plant still relies on the soil N-pool for half of its N-requirements. The Ndfa value obtained in our work is similar to that obtained for common bean by other authors, and can be considered low compared to other legumes (Guinet et al., 2018). These latter authors assigned the Ndfa values to the different ability among legume crop species to take up inorganic N from the soil. In the case of *P. vulgaris*, a high inorganic N uptake combined with relatively low values of %Ndfa maximizes N use efficiency (NUE) in soils with relatively high N-levels (a legacy owing to applications of fertilizer to previous seasons non-legume crops), which in turn reduces the risk of nitrogen leaching from the soil.

We then hypothesized that the superior effect of Rlp LCS0306 on the grain yield could be explained by its gene assortment, as it contains a large repertoire of secretion systems as well as the genes involved in an efficient symbiosis with *Phaseolus*. Apart from characterizing the N-fixing genetic machinery of Rlp LCS0306, the genomic analysis has also revealed other data of interest that taken together help to explain its superiority compared to strains Re CFN42^T^ and Rp ATCC 14482^T^. Despite having a genomic backbone homologous to the biovar viciae, Rlp LCS0306 contains the symbiotic repertoire of Re CFN42^T^. Rhizobial genomes are extremely variable (MacLean et al., 2007), with large replicons called chromids that appear to contain genus-specific genes in *Rhizobium*, *Ensifer* and *Agrobacterium* (Harrison et al., 2010) and secondary replicons, like symbiotic plasmids, that are generally more genetically diverse between strains than the primary chromosome (Galardini et al., 2013). Indeed, the largest contig in LCS0306 (NODE_1) shows a high degree of synteny compared to pRlvA chromid in Rlv UPM791 (as shown in Figure 4), which was in turn highly similar to pRL12 in Rlv 3841 (Sánchez-Cañizares et al., 2018). In general, the Rlp LCS0306 genome sequence showed ANI values above 95% when compared with Rlv UPM791 (Table 4), indicating that they share most of their genome content. For example, as with Rlv UPM791, LCS0306 only harbors an ortholog of the type I PHB synthase, *phbC1*, required for free-living poly-β-hydroxybutyrate (PHB) biosynthesis, a carbon polymer that seems to play a role during root infection and invasion (Trainer and Charles, 2006). Strain Rlp LCS0306 also harbors the two *fnrN* copies controlling the expression of the *fixNOQP* genes present in Re CFN42^T^ and Rlv UPM791 (Colombo et al., 2000; Lopez et al., 2001). Strain Rlp LCS0306 has three *nodD* copies, as has the p42d symbiotic plasmid from Re CFN42^T^, again highlighting the commonalities with the *R. etli* symbiotic plasmid. *NodD* regulates the expression of the *nodABCFE* cluster and, therefore, it is involved in Nod factor production. Similarly, five *nodD* reiterations were found in *R. tropici* CIAT899, necessary to engage the symbiont in nodulation with different legume species (Del Cerro et al., 2015). These *nodD* reiterations were also present in various different N-fixing rhizobial strains from *P. vulgaris*, suggesting a potential role in host range (Peralta et al., 2016). Sequence heterogeneity within p42d already suggested extensive genomic rearrangements, recombination rates, lateral transfer, and relaxation or intensification of selective pressures (González et al., 2003). This might have been the case in Rlp LCS0306 at the genome level, incorporating all those features that might impact positively on its competitiveness and symbiotic performance, thus resulting in a strain with outstanding agronomic properties.

### Effect of the Formulation

Once the superior behavior of Rlp LCS0306 was reinforced by its genomic potential, in order to design an inoculant based on this elite strain, the next step was to determine the optimal formulation to be applied as an inoculant under field conditions. Compared to the control formulation based on perlite, the PB formulation based on perlite and biochar produced a significantly higher number of pods per plant (14.48 versus 13.40 in the control) and also a significantly higher grain yield (3640 kg ha^–1^ versus 3165 kg ha^–1^ in the control). Interestingly, this was not accompanied with an increase either in the Ndfa (%), N-fixed in kg ha^–1^, nodule number, or nodule biomass compared to the other formulations used in the experiment. Thus, the improvement in field performance of the PB formulation suggests that it is related to the plant growth promoting effect assigned to biochar (Yang et al., 2019), rather than to a direct effect on the strain performance as a consequence of the formulation. The plant growth promoting effect of biochar can be explained in terms of hormone analogs contained within it (Graber et al., 2010), inducing the expression of certain genes related to plant growth (Huang et al., 2015; Mehari et al., 2015; Yang et al., 2019).

## Conclusion

The results obtained in this study explained the success under field conditions of an outstanding inoculant for common bean based on the autochthonous strain Rlp LCS0306 when it has been appropriately formulated, in terms of its symbiotic performance, genomic features and agronomic traits. Overall, the superior performance of strain Rlp LCS0306 appears to be due to the combination of different modes of action, which together produced a significantly higher grain yield and a high rate of recovery from nodules, compared to the resident rhizobia and to other common bean-nodulating strains like Re CFN42^T^ and Rp ATCC 14482^T^. From an agronomic perspective, Rlp LCS0306 is both a highly efficient N-fixer, which is competitive against the native rhizobia, as well as providing common bean with a stimulus to enhance its NUE. From the genomic perspective, the competitive behavior could be explained by its broad metabolic capacities and the large variety of its secretion systems. On the other hand, the enhanced yield obtained in the field with Rlp LCS0306 could be partially explained to some extent in terms of Ndfa (%) and nodule biomass. However, there may be other factors contributing to the superiority of LCS0306 in field trials, potentially derived from its gene assortment, as the strain harbors a *R. leguminosarum* scaffold with a symbiotic plasmid characteristic of strains nodulating *Phaseolus*, containing the genes required for an efficient symbiosis. Rlp LCS0306 has evolved in the local conditions of northern central plateau in Spain, and it has gathered several genomic characteristics as enumerated above, putatively involved in its adaptation to such local conditions. The ensemble of its diverse genomic characteristics, rather than a specific characteristic itself, seems to contribute to the superior performance of Rlp LCS0306 in the field. To date, cultivation of common bean with the available commercial inoculants has resulted in a suboptimal nodulation and BNF, as this crop is not native to Europe. This study constitutes the first evidence of a native inoculant enhancing BNF and grain yield in common bean in Spain, stressing its economic value for the future sustainable cultivation of this important crop.

## Data Availability Statement

The datasets generated for this study can be found in the NCBI BioProject no. PRJNA552714.

## Author Contributions

RP-B executed the field trial, collected the data and wrote the manuscript. CS-C analyzed the genomic data and wrote the manuscript. EJ worked on the general structure and the integration of the different parts. FG-A designed the field trial, analyzed the data, and wrote the manuscript.

## Conflict of Interest

The authors declare that the research was conducted in the absence of any commercial or financial relationships that could be construed as a potential conflict of interest.
