# Supplementary material for: Formulation of a Highly Effective Inoculant for Common Bean Based on an Autochthonous Elite Strain of Rhizobium leguminosarum bv. phaseoli, and Genomic-Based Insights Into Its Agronomic Performance
Source: Front Microbiol. 2019 Dec 17;10:2724. doi: 10.3389/fmicb.2019.02724 (PMC6927923; doi:10.3389/fmicb.2019.02724)
Supplement: Supplementary file 1 [file Data_Sheet_1.docx]

**Table S1**

Composition of the compost used as carrier

| Parameter | Value |
| --- | --- |
| Ca (mg/kg) | 19.95 |
| Cd (mg/kg) | 0.48 |
| Cr (mg/kg) | 21.81 |
| Cu (mg/kg) | 0.23 |
| Fe (mg/kg) | 21.65 |
| Hg (mg/kg) | 0.11 |
| K (mg/kg) | 12.13 |
| Mg (mg/kg) | 18.34 |
| Mn (mg/kg) | 42.08 |
| N Kjeldahl (%) | 1.80 |
| Na (mg/kg) | 15.26 |
| Ni (mg/kg) | 10.32 |
| P (mg/kg) | 14.95 |
| Pb (mg/kg) | 6.08 |
| Zn (mg/kg) | 28.29 |
| Oxidizable organic carbon (%) | 21.49 |
| Organic Matter (%) | 48.01 |
| pH (soil:water) | 6.91 |
| C/N Ratio | 15.49 |
| Electrical Conductivity (ds/m) | 2.07 |

**Table S2**

Climatic and edaphic conditions corresponding to the field experiments during 2017 and 2018. The climatic data were recorded at the León - Virgen del Camino provincial meteo station.

| Date | Temperatures (°C)* | | | | | Monthly rainfall (mm) |
| --- | --- | --- | --- | --- | --- | --- |
| 2017 | Hmax (ºC) | Havg (ºC) | Tavg (ºC) | Lavg (ºC) | Lmin (ºC) |  |
| May | 24.1 | 21.1 | 14.8 | 8.5 | -2.0 | 69.3 |
| June | 35.4 | 27.0 | 20.0 | 12.9 | 7.0 | 23.0 |
| July | 33.6 | 28.4 | 20.3 | 12.2 | 6.6 | 2.1 |
| August | 34.7 | 27.8 | 20.1 | 12.3 | 5.5 | 9.2 |
| September | 29.9 | 23.5 | 16.0 | 8.5 | 3.3 | 3.3 |
| October | 30.5 | 22.7 | 15.1 | 7.6 | 2.5 | 14.6 |
| 2018 |  |  |  |  |  |  |
| May | 24.1 | 19.0 | 12.7 | 6.5 | -1.5 | 72.7 |
| June | 32.9 | 23.6 | 17.5 | 11.4 | 6.3 | 111.3 |
| July | 29.8 | 25.8 | 19.2 | 12.5 | 9.5 | 57.9 |
| August | 35.3 | 29.3 | 20.7 | 12.1 | 6.6 | 2 |
| September | 31.2 | 27.1 | 19.6 | 12.0 | 5.8 | 14.1 |
| October | 27.1 | 18.1 | 11.8 | 5.5 | -0.4 | 27.1 |

| Year | | 2017 | 2018 |
| --- | --- | --- | --- |
| Location | | León EIAF | Oteruelo |
| Latitude | | 42°34'59.3"N | 42°34'56"N |
| Longitude | | 5°35'32.0"W | 5°36'40"W |
| Texture (%) | Sand | 24 | 22 |
|  | Silt | 42 | 34 |
|  | Clay | 34 | 44 |
| pH 1:2 | (soil:water) | 6.94 | 7.15 |
| Electric conducitiviy | (dS/m) | 0.16 | 0.14 |
| Organic matter (%) | | 3.56 | 2.34 |
| Total | nitrogen** (%) | 0.22 | 0.14 |
| Ratio C/N | | 9.22 | 9.96 |
| Lime | (%) | negligible | negligible |
| P -Olsen | (mg kg^-1^) | 17.28 | 21.13 |
| K | (cmol(+) kg^-1^) | 0.23 | 0.30 |
| Ca | (cmol(+) kg^-1^) | 17.47 | 15.85 |
| Mg | (cmol(+) kg^-1^) | 2.46 | 3.18 |
| Na | (cmol(+) kg^-1^) | 0.09 | 0.17 |
| Nodulating rhizobia count (MPN*** | Rhizobia g soil^-1^ | 5.8 x 10^3^ | 1 x 10^4^ |

* Hmax: maximum high temperature) (ºC); Havg: average high temperature (ºC); Tavg: average mean temperature (ºC); Lavg: average low temperature (ºC); Lmin: minimum low temperature (ºC); R: monthly precipitation (mm)

**Total N: organic + nitric + ammonia nitrogen.

*** Most Probable Number

**Table S3**

Raw data from δ^15^N_AIR_ (‰) values obtained for the common bean plants and the reference plants (*Sinapis arvensis* L., *Chenopodirum album* L. and *Oxalis corniculata* L., one third in weight from each).

| Treatment | Block | Year 2017 | | Year 2018 | |
| --- | --- | --- | --- | --- | --- |
|  |  | d^15^N_common bean_ | d^15^N_reference plants_ | d^15^N_common bean_ | d^15^N_reference plants_ |
| Negative control | 1 | 3.4 | 9.7 | 5.1 | 8.1 |
| Negative control | 2 | 4.3 | 9.7 | 4.9 | 8.1 |
| Negative control | 3 | 3.8 | 9.7 | 4.7 | 8.1 |
| N fertilizedsed non-inoculated control | 1 | 4.1 | 9.7 | 4.8 | 8.1 |
| N fertilizedsed non-inoculated control | 2 | 3.8 | 9.7 | 5.0 | 8.1 |
| N fertilizedsed non-inoculated control | 3 | 4.0 | 9.7 | 5.8 | 8.1 |
| Rlp LCS0306 (Co) | 1 | 3.4 | 9.7 | 5.1 | 8.1 |
| Rlp LCS0306 (Co) | 2 | 3.1 | 9.7 | 4.1 | 8.1 |
| Rlp LCS0306 (Co) | 3 | 3.2 | 9.7 | 4.1 | 8.1 |
| Rlp LCS0306 (CC) | 1 | 2.9 | 9.7 | 4.6 | 8.1 |
| Rlp LCS0306 (CC) | 2 | 2.6 | 9.7 | 4.0 | 8.1 |
| Rlp LCS0306 (CC) | 3 | 2.7 | 9.7 | 4.5 | 8.1 |
| Rlp LCS0306 (PB) | 1 | 2.5 | 9.7 | 4.7 | 8.1 |
| Rlp LCS0306 (PB) | 2 | 3.0 | 9.7 | 3.9 | 8.1 |
| Rlp LCS0306 (PB) | 3 | 2.6 | 9.7 | 4.1 | 8.1 |
| Rlp LCS0306 (perlite) | 1 | 2.2 | 9.7 | 4.6 | 8.1 |
| Rlp LCS0306 (perlite) | 2 | 2.5 | 9.7 | 4.4 | 8.1 |
| Rlp LCS0306 (perlite) | 3 | 2.8 | 9.7 | 3.8 | 8.1 |
| Re CFN42T (perlite) | 1 | 3.3 | 9.7 | 5.1 | 8.1 |
| Re CFN42T (perlite) | 2 | 3.6 | 9.7 | 5.0 | 8.1 |
| Re CFN42T (perlite) | 3 | 3.5 | 9.7 | 4.2 | 8.1 |
| Rp ATCC 14482T (perlite) | 1 | 2.8 | 9.7 | 4.7 | 8.1 |
| Rp ATCC 14482T (perlite) | 2 | 3.2 | 9.7 | 4.5 | 8.1 |
| Rp ATCC 14482T (perlite) | 3 | 3.3 | 9.7 | 4.1 | 8.1 |

**Table S4**

Mean squares corresponding to the combined ANOVA of the dependent variables related to nodulation, nitrogen fixation, yield, yield components and harvest index, collected in the field trial trial. Two different ANOVA were carried out, the first analysing the effect of the **inoculation** with different rhizobia strains (*R. leguminosarum* bv. phaseoli LCS0306, *R. phaseoli* ATCC 14482^T^ and *R. etli* CFN 42^T^) plus two uninoculated controls, one of them fertilised with mineral nitrogen. The second ANOVA analyses the effect of the **formulation** of the *R. leguminosarum* bv. phaseoli LCS0306 strain, using perlite as control (see text for more details). Significance levels: *** p≤0.001; ** 0.001<p≤0.01; *0.01<p≤0.05; ns not significant.

| Treatments  analysed | Source of variation | DF | Number of nodules per plant | Dry nodule biomass (g per plant) | Dry aerial biomass (kg/ha) | Aerial biomass N content (%) | Ndfa (%) | N fixed (kg/ha) | Soil N uptake (kg/ha) | Grain yield  (air dried)^1^ (kg/ha) | Pods per plant | Seeds per pod | 100-seeds weight  (dry) (g) | Harvest index |
| --- | --- | --- | --- | --- | --- | --- | --- | --- | --- | --- | --- | --- | --- | --- |
| **Inoculation** treatment (rhizobia strain plus controls) | Analysis of the repetition (R) | 2 | 42.146 ns | 0.014 ns | 172902.437 ns | 0.092 ns | 5.896 ns | 58.557 ns | 95.106 ns | 69220.23 ns | 1.446 ns | 0.039 ns | 7.833 ns | 1.192 ns |
|  | Analysis of the treatment | | | | | | | | | | | | | |
|  | Treatment (T) | 4 | 63.211ns | 0.421*** | 3808183.48*** | 0.046 ns | 92.116** | 1034.93*** | 615.937*** | 1560018 *** | 17.086 *** | 0.324 *** | 11.147 ns | 54.522 * |
|  | T x Year | 5 | 8.021 ns | 0.007 ns | 100139.655 ns | 0.053 ns | 3.283 ns | 99.616 ns | 35.231 ns | 593239.9*** | 4.379** | 0.355** | 65.791*** | 123.941*** |
|  | T x R | 8 | 75.765 ns | 0.026 ns | 63948.271 ns | 0.064 ns | 7.348 ns | 55.184 ns | 45.77 ns | 4668.15 ns | 0.402 ns | 0.01 ns | 3.442 ns | 5.781 ns |
| **Formulation** for the LCS0306 strain (Formulation with Perlite was the control) | Analysis of the repetition (R) | 2 | 8.667 ns | 0.001 ns | 118374 ns | 0.053 ns | 15.167 ns | 32.596 ns | 90.791 ns | 87751.5 ns | 0.353 ns | 0.062 ns | 4.314 ns | 5.399 ns |
|  | Analysis of the treatment | | | | | | | | | | | | | |
|  | Treatment (T) | 3 | 55.667 ns | 0.031 ns | 429316.819 ns | 0.054 ns | 23.667 ns | 350.339 * | 297.946 * | 292425.042 ** | 1.373 ** | 0.061 ns | 4.095 ** | 12.332 ns |
|  | T x Year | 3 | 8.944 ns | 0.007 ns | 162426.264 ns | 0.035 ns | 2.5 ns | 44.112 ns | 8.092 ns | 13856.931 ns | 0.076 ns | 0.089 ns | 5.338 ** | 6.563 ns |
|  | T x R | 6 | 129.667 * | 0.044 ns | 116453.444 ns | 0.024 ns | 3.167 ns | 42.927 ns | 23.648 ns | 33450.167 ns | 0.284 ns | 0.028 ns | 2.144 * | 23.569 ns |

^1^ Corresponds to the commercial beans (11.57 % dry matter)

**Table S5**

Rlp LCS0306 genome statistics.

| Genome ID | Rlp LCS0306 |
| --- | --- |
| Total length (bp) | 7,395,396 |
| GC (%) | 60.72 |
| N50 | 340,266 |
| N75 | 161,667 |
| L50 | 7 |
| L75 | 14 |
| # N's | 0.00 |
| # N's per 100 kbp | 0.00 |
| # contigs (>= 0 bp) | 135 |
| # contigs (>= 1000 bp) | 58 |
| Total length (>= 0 bp) | 7,395,396 |
| Total length (>= 1000 bp) | 7,360,538 |
| # contigs | 71 |
| Largest contig | 929,560 |
| Genes (total) | 7,172 |
| CDSs (total) | 7,115 |
| Genes (coding) | 6,906 |
| Genes (RNA) | 57 |
| rRNAs | 2, 1, 4 (5S, 16S, 23S) |
| tRNAs | 46 |
| ncRNAs | 4 |
| Pseudo Genes (total) | 209 |

**Table S6**

LCS0306 search for Cluster of Orthologous Groups (COG). The values reflect the number of protein families, coverage and abundance in LCS0306 genome as a result of the comparison of LCS0306 functional annotation to protein sequences encoded in complete genomes from the COG protein database (WebMGA server).

| #Class | No families | Coverage | Abundance | Description |
| --- | --- | --- | --- | --- |
| J | 245 | 0.0082 | 0.1242 | Translation, ribosomal structure and biogenesis |
| A | 25 | 0 | 0 | RNA processing and modification |
| K | 231 | 0.0087 | 0.0567 | Transcription |
| L | 238 | 0.0126 | 0.2313 | Replication, recombination and repair |
| B | 19 | 0 | 0 | Chromatin structure and dynamics |
| D | 72 | 0 | 0 | Cell cycle control, cell division, chromosome partitioning |
| Y | 2 | 0 | 0 | Nuclear structure |
| V | 46 | 0 | 0 | Defense mechanisms |
| T | 152 | 0 | 0 | Signal transduction mechanisms |
| M | 188 | 0 | 0 | Cell wall/membrane/envelope biogenesis |
| N | 96 | 0 | 0 | Cell motility |
| Z | 12 | 0 | 0 | Cytoskeleton |
| W | 1 | 0 | 0 | Extracellular structures |
| U | 158 | 0 | 0 | Intracellular trafficking, secretion, and vesicular transport |
| O | 203 | 0.0049 | 0.0298 | Posttranslational modification, protein turnover, chaperones |
| C | 258 | 0.0155 | 0.2372 | Energy production and conversion |
| G | 230 | 0.0087 | 0.0966 | Carbohydrate transport and metabolism |
| E | 270 | 0.0074 | 0.0420 | Amino acid transport and metabolism |
| F | 95 | 0.0105 | 0.0341 | Nucleotide transport and metabolism |
| H | 179 | 0 | 0 | Coenzyme transport and metabolism |
| I | 94 | 0 | 0 | Lipid transport and metabolism |
| P | 212 | 0.0047 | 0.0210 | Inorganic ion transport and metabolism |
| Q | 88 | 0 | 0 | Secondary metabolites biosynthesis, transport and catabolism |
| R | 702 | 0.0028 | 0.1419 | General function prediction only |
| S | 1347 | 0.0007 | 0.0210 | Function unknown |
|  |  |  |  |  |
| TOTAL | 5163 | 0.0848 | 1.0358 |  |

**Table S7**

Use of carbon and nitrogen sources by reference *Rhizobium* strains and the autochthnous strain *R. leguminsarum* bv. phaseoli LCS0306. Characterisation and selection of rhizobia nodulanting common bean (*Phaseolus vulgaris* L.) in the P.G.I. “*Alubia de la Bañeza-León*”. Unpublished data from Mulas, D. 2010. PhD. Dissertation. University of León. Spain. 191 pp.

| **Carbon sources** | *R. phaseoli* ATCC 14482^T^ | *R. leguminosarum* USDA 2370^T^ | *R etli* CFN42^T^ | *R. leguminsarum* bv. phaseoli LCS0306 |
| --- | --- | --- | --- | --- |
| D-rafinosa | + | + | + | + |
| Galactose | + | + | + | + |
| Maltose | + | + | + | + |
| D-celobiose | + | + | + | + |
| Melibiose | + | + | + | + |
| D-sucrose | + | + | + | + |
| D-salicine | - | - | - | + |
| D-trehalose | + | + | + | + |
| L-ramnose | + | + | + | + |
| L-sorbose | + | + | + | + |
| D-manose | + | + | + | + |
| Fructose | + | + | + | + |
| Xilose | + | + | + | + |
| Melecitose | - | + | + | + |
| Xilitol | + | + | + | + |
| D-sorbitol | + | + | + | + |
| Meso-eritritol | + | + | - | + |
| Inositol | + | + | + | + |
| Manitol | - | + | + | + |
| Na Piruvate | + | + | + | + |
| Na Glucuronate | - | - | - | - |
| Na Propionate | + | - | - | + |
| Na Gluconate | - | - | - | - |
| Na Citrate | - | + | + | + |
|  |  |  |  |  |
| **Nitrogen sources** |  |  |  |  |
| L-serina | - | - | + | + |
| DL-valina | - | - | + | + |
| L-alanina | - | + | + | + |
| L-prolina | + | + | + | + |
| Betaína | + | + | + | + |
| L-arginina | - | - | - | - |
| L-lisina | - | - | - | - |
| L-histidina | + | - | + | + |
| Sarcosina | + | + | + | + |
| Aspartato Mg | + | + | + | + |
| Glutamato Na | + | - | + | + |

**Table S8**

Distribution of genes linked to competitiveness in Rlp LCS0306 genome compared to reference *Rhizobium* strains. Homologue genes are indicated in the corresponding column.

| Gene function | Gene name | Locus_tag | Contig | *R. leguminosarum* UMP791 | *R. phaseoli* ATCC14482^T^ | *R.etli*  CFN42^T^ |
| --- | --- | --- | --- | --- | --- | --- |
| ABC transporters | *aapJQMP*  *teuBAC1C2*  *nocR,*  *nocQCT* | FML87_20425-  FML87_20440  FML87_29775 -  FML87_29760  FML87_29495  FML87_29500-  FML87_29510 | Node 9  Node 19*  Node 19* | RLV_4523- RLV_4520 |  | RHE_PD00128-RHE_PD00131  RHE_PD00181  RHE_PD00180-  RHE_PD00178 |
| Motility | *motA*  *motB*  *flg* and *fli* genes | FML87_25710  FML87_25610  FML87_25750-  FML87_25545 | Node 13  Node 13 | RLV_3097  RLV_3117  RLV_3089- RLV_3131 |  |  |
| Chemotaxis | *cheAWRBYD* (Che1 cluster) | FML87_25785-  FML87_25760 | Node 13 | RLV_3082- RLV_3087 |  |  |
| EPS biosynthesis and transport | *pssSRMLKJIFCDE*  *pssTONP*  *prsED* | FML87_17650-  FML87_17705  FML87_17755-  FML87_17740  FML87_17715-  FML87_17720 | Node 7  Node 7  Node 7 | RLV_5915-  RLV_5925  RLV_5932- RLV_5935  RLV_5927  RLV_5928 |  |  |
| Peptidoglycan biosynthesis | *ftsI murEF mraY murD ftsW murGBC,* | FML87_12315-  FML87_12275 | Node 4 | RLV_5562- RLV_5560 | EFD56_29325- EFD56_29285 |  |
| RSI bacterial invasion switch | *pckA chvIG*  *hprK manX npr* | FML87_28000-  FML87_27975 | Node 16 | RLV_7039- RLV_7044 |  |  |
| PHB | *phbC1* | FML87_20625 | Node 9 | RLV_4485 |  |  |
| Type III secretion system (T3SS) | *rhcJ, rhcLNQRSTU, hrpW, rhcVD, rhC1O* | FML87_34175-  FML87_34265 | Node 33 |  |  | RHE_PD00051-  RHE_PD00067 |
| Type IV secretion system - pilus (T4SS) | *virB1-virB11* | FML87_29685 –  FML87_29510 | Node 19 | RLV_0329-  RLV_0340 |  |  |
| Type IV secretion system – *tra* conjugal system (T4SS) | *traCDG*  *traA-traFBHMR* | FML87_29555 - FML87_29565  FML87_29550 - FML87_29525 | Node19* |  |  | RHE_PD00167- RHE_PD00175 |
| Type VI secretion system (T6SS) | *tssABC Hcp tssEFGI*  *tssKLM* | FML87_29395 - FML87_29430  FML87_29375 - FML87_29360 | Node 19 |  | EFD56_30825- EFD56_30795 |  |
| Quorum sensing (bacteriocin production) | *cinRIS* | FML87_12665-  FML87_12675 | Node 4 | RLV_5631-  RLV_5632 |  |  |

*Note that Node_19 aligns with the symbiotic plasmid of *R.etli* CFN42 ^T^ (p42d)

**Table S9**

Distribution of genes contributing to symbiosis in Rlp LCS0306 genome compared to reference *Rhizobium* strains. Homologue genes are indicated in the corresponding column.

| Gene function | Gene name | Locus_tag | Contig | *R. leguminosarum* UMP791 | *R. phaseoli*  ATCC14482^T^ | *R.etli*  CFN42^T^ |
| --- | --- | --- | --- | --- | --- | --- |
| Nitrogenase | *rpoN-nifUSW*  *nifAB fdxN nifZT*  *nifDKEX fdxB* | FML87_33610 –  FML87_33625  FML87_33650 -  FML87_33670  FML87_34560 –  FML87_34585 | Node 30  Node 30  Node 36 |  | EFD56_29645- EFD56_29620  EFD56_29595-EFD56_29575 | RHE_PD00218- RHE_PD00222  RHE_PD00228- RHE_PD00231  RHE_PD00307-RHE_PD00302 |
| Nitrogen fixation | *fixNOQPGHIS* (FIX1 region)  *fixABCX* (FIX2 region) | FML87_34615-  FML87_34655  FML87_33630 –  FML87_33645 | Node 36  Node 30 | RLV_1827-RLV_1834 |  | RHE_PD00296- RHE_PD00289  RHE_PD00224- RHE_PD00227 |
| Nodulation | *nodA*  *nodBCSUIJ*  *nodD1*  *nodD2*  *nodD3* | FML87_35315  FML87_34775-  FML87_34745  FML87_34730  FML87_34950  FML87_34945 | Node 47  Node 37  Node 37  Node 39  Node 39 |  |  | RHE_PD00310  RHE_PD00282- RHE_PD00277  RHE_PD00275  RHE_PD00316  RHE_PD00318 |
| Regulation | *fnrN1*  *fnrN2*  *rosR* | FML87_28695  FML87_33600  FML87_24335 | Node 17  Node 30  Node 12 | RLV_5077  RLV_1980  RLV_3788 |  | RHE_CH02479  RHE_PD00216  RHE_CH01249 |
